# Misleading Population Estimates: Biases and Consistency of Visual Surveys and Matrix Modelling in the Endangered Bearded Vulture

**DOI:** 10.1371/journal.pone.0026784

**Published:** 2011-10-21

**Authors:** Antoni Margalida, Daniel Oro, Ainara Cortés-Avizanda, Rafael Heredia, José A. Donázar

**Affiliations:** 1 Bearded Vulture Study and Protection Group, El Pont de Suert, Lleida, Spain; 2 Division of Conservation Biology, Institute of Ecology and Evolution, University of Bern, Bern, Switzerland; 3 Institut Mediterrani d’Estudis Avançats IMEDEA, CSIC-UIB, Esporles, Spain; 4 Department of Ecological Modelling, Helmholtz Centre for Environmental Research-UFZ, UFZ, Leipzig, Germany; 5 Department of Conservation Biology, Estación Biológica de Doñana, CSIC, Sevilla, Spain; 6 Somió, Gijón, Spain; University of Bern, Switzerland

## Abstract

Conservation strategies for long-lived vertebrates require accurate estimates of parameters relative to the populations' size, numbers of non-breeding individuals (the “cryptic” fraction of the population) and the age structure. Frequently, visual survey techniques are used to make these estimates but the accuracy of these approaches is questionable, mainly because of the existence of numerous potential biases. Here we compare data on population trends and age structure in a bearded vulture (*Gypaetus barbatus*) population from visual surveys performed at supplementary feeding stations with data derived from population matrix-modelling approximations. Our results suggest that visual surveys overestimate the number of immature (<2 years old) birds, whereas subadults (3–5 y.o.) and adults (>6 y.o.) were underestimated in comparison with the predictions of a population model using a stable-age distribution. In addition, we found that visual surveys did not provide conclusive information on true variations in the size of the focal population. Our results suggest that although long-term studies (i.e. population matrix modelling based on capture-recapture procedures) are a more time-consuming method, they provide more reliable and robust estimates of population parameters needed in designing and applying conservation strategies. The findings shown here are likely transferable to the management and conservation of other long-lived vertebrate populations that share similar life-history traits and ecological requirements.

## Introduction

The estimation of the non-breeding fraction of wild vertebrate populations is of great interest to ecologists and conservationists because these individuals represent a buffer that can allow populations to survive stochastic environmental and demographic phenomena [1]–[5]. In addition, changes in the non-breeding population's size and age structure may serve as accurate early-warning signals previous to the decline of the breeding population [6], [7].

Thus, for conservation purposes, it is essential to take into account the existing relationship between settlement-breeding areas as a consequence of the influence of the dynamics within entire populations [8]–[11]. Estimates of the non-breeding fraction are difficult to ascertain because they are often “cryptic” in nature and are generally not detectable other than via long-term monitoring programmes or complex approximations based on molecular techniques [12]. This question is accentuated in the case of long-lived vertebrates characterized by delayed reproduction, which ensures that individuals are not recruited into the breeding population until they are a few years old. In the meantime, these individuals often lead dispersive life-styles not necessarily linked to their main breeding areas (i.e. the spatial segregation of non-breeding birds) [13], [14]. Although in raptors it is assumed that the non-breeding fraction of populations is mainly composed of juvenile individuals [15], [16], information regarding their age structure remains scarcely explored in the literature [11], [17].

The application of the results of direct observations and individual counts (hereafter, visual surveys) in determining population age structures and demographic trends is frequently used as a basis for conservation proposals to aid in the recovery of threatened vertebrates [18]–[21]. Particularly, the aggregation of individuals at migratory stopovers, roosting sites and at sites with an abundance of trophic resources (e.g., the so-called vulture restaurants) [22] has been used to determine the age structure of populations of birds with large but scattered populations that are difficult to monitor [23], [25]. Nevertheless, the probability of detecting individuals by means of visual surveys may be asymmetrical due to both the individual's behaviour and/or environmental variability [26]. To assess these potential biases it is necessary to compare results obtained from visual surveys with precise estimates derived from matrix population models. To date, very few such analyses have been performed, probably due to the inherent difficulty in obtaining long-term monitoring data for long-lived vertebrate populations [27]–[30].

The bearded vulture (*Gypaetus barbatus*) is a long-lived territorial vulture species that is under threat in Europe (170 pairs in the European Union in 2010). Owing to its specialized diet based on the bones of wild and domestic ungulates [31], the conservation methods employed to ensure the recovery of the Pyrenean sub-population (145 pairs), the largest in Europe, are based on supplementary feeding (i.e. provision of sheep limbs and other bone matter in winter). As a result, almost all of the non-breeding population is concentrated around this network of feeding stations [32], [33]. These large aggregations of dozens of non-breeding birds make visual surveys feasible and the information thus obtained is one of the basic tools used by regional and state governments to draw up conservation strategies [34]. Apart from this, since the end of the 1980 s, the Pyrenean bearded vulture population has also been monitored using individual tagging, which has enabled demographic models based on capture-recapture and stochastic population modelling [35].

Taking advantage of this scenario, our main objective is to compare the population trend and age structure estimated on the basis of visual surveys with data derived from approximations reached via matrix population models. As a corollary, we examined the existence of age-related asymmetries in the use of supplementary feeding stations by bearded vultures.

## Materials and Methods

### Field procedures

During 1990–2006, between 9 and 13 (mean±sd: 11.5±1.5) supplementary feeding stations were surveyed annually. The annual visual surveys (mean±sd: 3.53±1.28) were carried out simultaneously at the end of winter (February-April), coinciding with the beginning of the breeding period (incubation and first weeks of chick-rearing) [36] and with the maximum numbers of birds at feeding stations owing to food shortages in the field [32], [33]. A minimum of two experienced observers equipped with 20−60×telescopes and binoculars monitored each supplementary feeding station between 09:00–16:00 h from dominant vantage points. Birds were identified individually on the basis of plumage characteristics and patterns recorded on standardized data sheets.

Accordingly, we considered the following age categories [32], [37]:

i): <2 years (immature): robust silhouette in flight, head and shoulders black, but back, chest, belly and legs brown.

ii): 3–5 years (subadult): more stylized flight silhouette than immature birds, with moulted primary and/or secondary flight feathers; feathers on face, head and neck beginning to turn white; the back is still brown and the chest, belly and legs are also still brownish, although the orange tones are beginning to appear.

iii): >6 years (adult): stylized, svelte silhouette in flight; head, chest and belly colour vary from white to orange; the back is now slate grey or black with an obvious contrast with the head.

In addition, the existence of wing-tags or rings was also recorded [35]. On the basis of all of this information and for each day of census the maximum number of birds belonging to the three age categories was estimated.

### Parameter estimations

We built an age-structured model that was initially used for projecting the evolution of the bearded vulture population in the Pyrenees [35] on the basis of tagged individuals. Demographic and environmental stochasticities were introduced into the model, as well as density-dependence on fertility [38]. We assumed that recruitment started at the age of six and was completed at 12 (i.e. a total of 12 age-classes were considered [see also 35]); survival probabilities decreased with time in line with capture-recapture estimates [35]. The 12 age-classes considered by the model were easily accommodated to the three age classes obtained from the counts. The model considered a post-breeding census and was a one-sex (female) model, because we had no evidences for sex-dependent parameters [35]. Initial conditions in year *t* = 1 (1990 in our case) were set to include the number of females in each age class. This number was estimated as follows: a deterministic model using average demographic estimates was run and to start such run the total number of females (breeders and non-breeders) was divided by the number of age-classes and such number was assigned equally to each age class. The resulting scaled right eigenvector of the matrix (i.e. the stable stage distribution, see below) was used to multiply the estimated number of females for each age-class in the population in *t* = 1 to set the initial conditions of the stochastic simulations. Given that the model had an age structure we were able to calculate the mean scaled population structure, i.e. the abundances of each age class relative to the abundance of the first age-class [39], once the model converged and the population tended asymptotically to that age-structure and growth rate [40]. The right eigenvector of the population matrix *W* is the stable stage distribution; the proportion of individuals in ages [1, 2, ….*i*] at time *t* is:



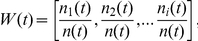
with *n_i_(t)* being the abundance of the oldest age class at time *t* and *n* the total population size. After a few generations (i.e. at demographic equilibrium) *W(t)* →*W*, i.e. the stable age distribution. Monte Carlo simulations were run for 23 years to cover the study period (1986–2008), and yearly estimation of population sizes obtained were compared it with the counts from visual surveysNote that such estimates of population size for each age-class were multiplied by two because the model yielded only the number of females, and counts included females and males.

### Data analyses

We used Generalized Linear Mixed Models (GLMM) [41], [42] to explore the relationship between the maximum observed abundance of bearded vultures and that predicted by the model (link function: logarithmic, error distribution: Poisson). Age was included as a factor with three levels corresponding to the above-described categories. ‘Year’ was fixed in the models as the random term to account for inter-annual variability [43], [44].

## Results

**Figure 1:**
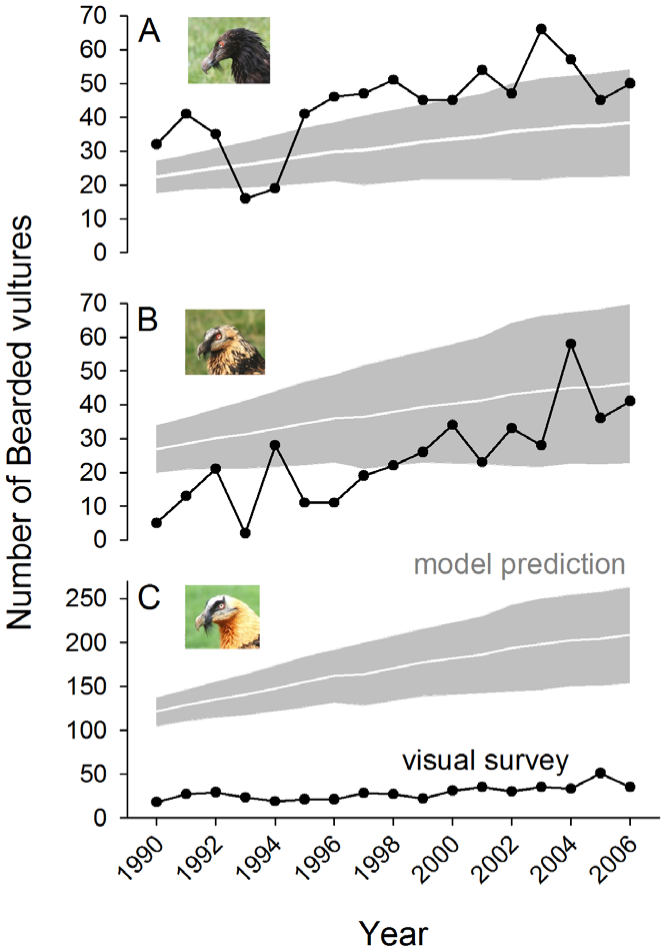
Annual variation in the estimates of a bearded vulture population through visual surveys (dark line) and population matrix-modelling approximations (white line with±sd in grey) for immature (<2 yrs old, A), subadult (3–5 yrs old, B) and adult (>6 yrs old, C) individuals. Note the different y-axis scales.

The maximum number of bearded vultures observed during the simultaneous visual surveys was significantly related to both the abundance predicted by the model (*F*_1, 31_ = 13.48, *p* = 0.0009) and the age of the individuals (*F*_2,31_ = 19.75, *p*<0.0001: adult< subadult<immature). These results initially seem to indicate that visual surveys reflected the real population trend (a progressive increase, Fig. 1). However, this relationship was only approximate: inter-annual variability was strong and visual surveys were not good at detecting finely tuned trends such as the slowing down of the population growth observed during the final years of the study (2004–2006, Fig. 1). In addition, visual surveys also overestimated the number of immature birds, but underestimated the numbers of subadults and adults with respect to the predictions of the population model using stable-age distribution (Fig. 1), being the mismatch for the adult age class extremely large.

## Discussion

Visual surveys at supplementary feeding stations did not reflect the structure of the bearded vulture population. Compared to the approximation derived from matrix modelling, the visual surveys significantly overvalued the size of the juvenile age class, underestimating the numbers of subadults and adults. Stable-age distribution from population modelling can be considered robust because many of the parameters of the model were reliable and age-structure is insensitive to additional mortality in all age-classes [35]. Consequently, visual surveys would seem to be inappropriate for assessing age-class structures in this bearded vulture population. The only other data available for the species were obtained by [18] in South Africa, where visual surveys estimated a population age-structure composed of 37% juveniles, 3% subadults and 60% adults. However, in this case the estimates were not made at feeding stations; no other data are available in the literature.

A possible explanation of the inflation observed in the juvenile age class could be related to the difficulties involved in identifying unmarked individualss, which could lead to repeated observations of the same bird at the feeding stations. Individuals of this age class have less experience in searching and obtaining food [45], [46] and so are highly dependent on predictable food sources and are much more regular at feeding stations. Thus, over 50 juveniles are sometimes observed at the same site on the same day [33], [35]. Aside from the inherent difficulties in individualizing a large number of birds, a single individual may visit more than one feeding station in a day (authors' unpubl. data), which would also help explain the overestimation.

Additionally, the results suggest that numbers of subadults and, above all, adult birds were underestimated in visual surveys. Lower numbers of adults may be related to reproductive duties [47], greater experience in searching for and finding food and special qualitative requirements (a need for small prey items with flesh during the first phases of the breeding period) [31]. All of these factors contribute to a lower dependence of territorial adults on supplementary feeding. Subadult birds are in the phase of territorial prospecting and mate-choice since pair-forming takes place at five-years old and territorial establishment at 6.5 years old [48].

Counts at fixed points (in our case feeding stations) may be biased by a number of factors [24], [49]. Thus, winter counts could increase the number of non-experienced individuals (juvenile age-class) observed, since they are more dependent on resources that are predictable in space and time during periods of adverse weather. At high bird densities (as occurs at feeding stations) observers may be overwhelmed by the numbers of birds to be identified and counted, thus hampering the separation of previously recorded individuals [24]. In addition, other factors such as food availability and the presence of competitors may also seriously affect the presence and abundance of individual birds [43], [50].

In conclusion, visual surveys are not useful for detecting small-scale variations in population sizes or for establishing the real age structure of a population. From a conservation point of view, this is potentially quite serious since the strategies implemented by administrations are often scarcely flexible and based on information that is difficult to keep up-to-date for proper adaptive management. Thus, visual surveys were likewise unable to detect a tendency towards stabilization in the study population size during 2004–2006. Taking into account the fact that in long-lived species population growth is generally highly sensitive to changes in adult survival [13,14,51, but see 52], the evaluations of population trends based on visual surveys tend to be over-optimistic and slow to detect regressive tendencies. In contrast, counts at feeding stations can provide useful information if visual surveys are focused on the monitoring of marked birds. When demographic data (i.e., capture-recapture) and counts (i.e., visual surveys) are both available, they can be analysed in conjunction using integrated population models (see [53] and references therein). These procedures have many advantages in terms of parameter estimations and accuracy, allowing the estimation of more demographic quantities, being the parameter estimates more precise. This also would allow including all sources of uncertainty due to process variability and the sampling processes [53]. In the case of the bearded vulture in the Pyrenees, capture-recapture approaches have sounded the alarm regarding the conservation status of the largest European population of this species [35], which suffers high mortality rates due to poisoned baits [54] and lead poisoning [55]. Likewise, the importance of demographic studies for planning conservation strategies becomes even greater in light of the fall in fecundity as a consequence of the density-dependence phenomenon, the ingestion of drugs, and the increase in the number of polyandrous groups [38], [56], [57].

Our findings are likely transferable to the management and conservation of other long-lived vertebrate populations that share common life-history traits and ecological requirements. Thus, estimations of the size of “cryptic” fraction of the populations [12] may be misleading in those scenarios leading to the spatial concentration of individuals. Despite being a more time-consuming method and the fact that its robustness depends on the number of individuals marked, population matrix modelling based on capture-recapture procedures would seem to be the only reliable and robust way of assessing age-structure and thus enabling the basis of conservation measures on objective data [58]. Given that currently most vulture populations are reinforced by supplementary feeding programmes [59], [60] and that several estimates of declining populations are based on visual surveys [61], [62], conservationists and managers should take into account the biases that these rapid surveys entail.
